# Applications of Deep-Learning in Exploiting Large-Scale and Heterogeneous Compound Data in Industrial Pharmaceutical Research

**DOI:** 10.3389/fphar.2019.01303

**Published:** 2019-11-05

**Authors:** Laurianne David, Josep Arús-Pous, Johan Karlsson, Ola Engkvist, Esben Jannik Bjerrum, Thierry Kogej, Jan M. Kriegl, Bernd Beck, Hongming Chen

**Affiliations:** ^1^Hit Discovery, Discovery Sciences, Biopharmaceutical R&D, AstraZeneca, Gothenburg, Sweden; ^2^Department of Life Science Informatics, B-IT, Rheinische Friedrich-Wilhelms-Universität Bonn, Bonn, Germany; ^3^Department of Chemistry and Biochemistry, University of Bern, Bern, Switzerland; ^4^Quantitative Biology, Discovery Sciences, Biopharmaceutical R&D, AstraZeneca, Gothenburg, Sweden; ^5^Department of Medicinal Chemistry, Boehringer Ingelheim Pharma GmbH & Co. KG, Biberach an der Riss, Germany; ^6^Chemistry and Chemical Biology Centre, Guangzhou Regenerative Medicine and Health – Guangdong Laboratory, Guangzhou, China

**Keywords:** Artificial intelligence, deep learning, Chemogenomics, Large-scale data, pharmaceutical industry

## Abstract

In recent years, the development of high-throughput screening (HTS) technologies and their establishment in an industrialized environment have given scientists the possibility to test millions of molecules and profile them against a multitude of biological targets in a short period of time, generating data in a much faster pace and with a higher quality than before. Besides the structure activity data from traditional bioassays, more complex assays such as transcriptomics profiling or imaging have also been established as routine profiling experiments thanks to the advancement of Next Generation Sequencing or automated microscopy technologies. In industrial pharmaceutical research, these technologies are typically established in conjunction with automated platforms in order to enable efficient handling of screening collections of thousands to millions of compounds. To exploit the ever-growing amount of data that are generated by these approaches, computational techniques are constantly evolving. In this regard, artificial intelligence technologies such as deep learning and machine learning methods play a key role in cheminformatics and bio-image analytics fields to address activity prediction, scaffold hopping, *de novo* molecule design, reaction/retrosynthesis predictions, or high content screening analysis. Herein we summarize the current state of analyzing large-scale compound data in industrial pharmaceutical research and describe the impact it has had on the drug discovery process over the last two decades, with a specific focus on deep-learning technologies.

## Introduction

Digital data, in all shapes and sizes, are growing exponentially. According to the National Security Agency of the United States, the Internet is processing around 1.8 billion GB of data per day (Macarron et al., 2011). In 2011, digital information has grown nine times in volume in just 5 years (Mayr and Bojanic, 2009) and by 2020, its amount in the world is expected to reach 35 trillion GB (Borman, 1999). The recent development of deep learning and other artificial intelligence methods is fuelled by the desire to seek greater insight among the ever-increasing amount of data in several key industries and powered by technological advancements as in, for example, computer vision, natural language processing, internet of things (IoT), or computer hardware.

Over the past decade, there has been a remarkable increase in the amount of available compound activity, biomedical (Borman, 1999; Mayr and Bojanic, 2009; Schamberger et al., 2011), and genomics data (Guyer and Collins, 1995; Human Genome Project Results; Wilson and Nicholls, 2015) thanks to the rapid development of high-throughput screening (HTS) and gene sequencing technologies. Typically, databases in pharma companies contain around 1–4 million compounds with biological data for several thousands of biological end-points such as targets or activities in cellular assays. Furthermore, due to the increasing level of automation and standardization, larger data sets of consistent conditions have become available. All chemical compounds synthesized and/or extracted from publications represent around 96 million compounds (Kim et al., 2019). Even though only a small fraction of them have associated biological information (Wang et al., 2014; Kim, 2016), these chemogenomics data sets alone already represent a formidable task for predictive modelling work.

The usage of new automation technologies resulted in a large volume of data, which has promoted the usage of machine learning (ML) methods. ML methods such as support vector machine (SVM), random forest (RF), or neural networks (NNs) have been used for data modelling in cheminformatics and bioinformatics for a long time. Only recently, various deep learning methods have become more popular due to the availability of large-scale training sets and high-performance computer hardware. An important difference between deep learning and previous ML methods is the flexibility of NN architectures and input/output data structures in deep learning methods and the automated extraction of features from raw data representations. This flexibility allows to design models that fit to the characteristics of the prediction problem (Wu et al., 2018; Xiong et al., 2019; Yang et al., 2019). Some of the popular NN architectures include convolutional NNs, recurrent NNs, autoencoders, and fully connected deep NNs. These deep learning methods have been applied (Ramsundar et al., 2017; Chen et al., 2018) on aspects of compound activity prediction (Dahl et al., 2014; Ma et al., 2015; Koutsoukas et al., 2017), *de novo* molecular design (Brown et al., 2019), protein–ligand interaction prediction (Lenselink et al., 2017; Feinberg et al., 2018), predictive toxicity (Mayr et al., 2016), and reaction prediction (Segler and Waller, 2017b). In this review, we will provide an overview on various types of large-scale data sets that are available in pharmaceutical industry. Such data sets offer a wealth of information that are unavailable in the public domain and give rise to a broad range of applications. Furthermore, we will exemplify the applications of artificial intelligence, in particular deep-learning technologies, that are powered through these large data sets on various problems in drug discovery.

## Large-Scale Compound Data in Pharmaceutical Industry

**Figure 1:**
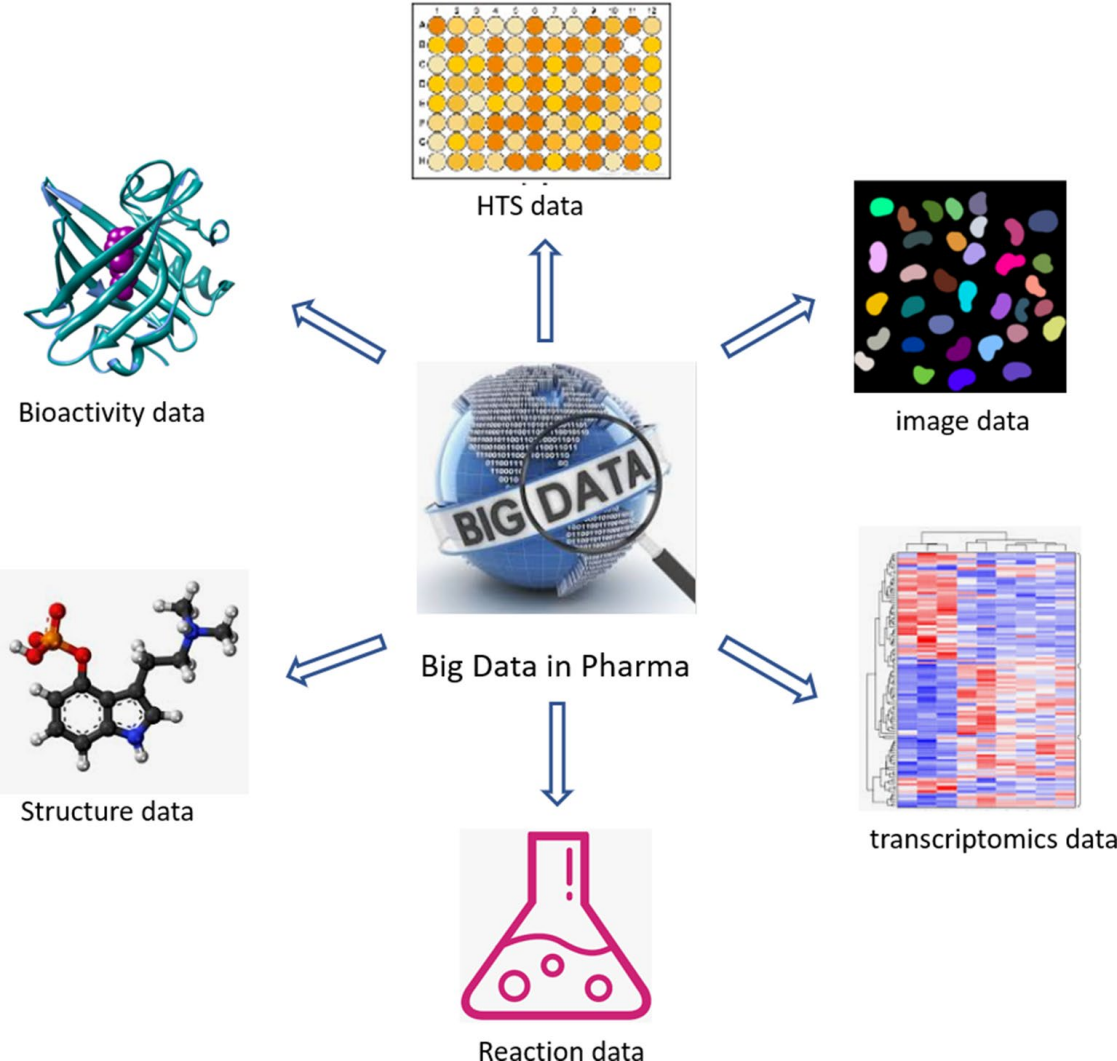
Different categories of large-scale compound data in industrial pharmaceutical research.

The past two decades have seen an acceleration of compound data generation in pharmaceutical industry driven by the technical advancement of HTS (Mayr and Bojanic, 2009; Macarron et al., 2011), parallel chemical synthesis (Borman, 1999), as well as the by the introduction of automation in sequencing and imaging. The various types of large-scale compound data in pharmaceutical research are illustrated in **Figure 1**. A small molecule database belongs to the core infrastructure of industrial pharma R&D in order to store the results of lead identification and optimization campaigns, which are used for, e.g., structure–activity–relationship (SAR) analyses. The typical size of a compound collection at major pharma companies ranges from 1 to 4 million compounds (Schamberger et al., 2011; Kogej et al., 2013). Compound activity data (including Administration Distribution Metabolism Excretion Toxicology (ADMET) end points) are the major part of the “Compound Data Estate” in pharmaceutical industry. Most of the SAR data come from the HTS campaigns carried out during the drug discovery projects, which typically comprise crude readouts generated from *in vitro* assays at single compound concentration—so called single-shot-potency—in the primary screening stage, and more accurate concentration response data (IC50s, EC50s, etc.) derived from multiple compound concentration experiments. Pharmaceutical databases allow for in-depth studies that may not be achievable with public data. Indeed, structuration and curation of private databases are done with the inclusion of concepts such as screening campaigns or lead optimization programs, which make possible a faster and easier analysis of high-quality data. Occasionally, the overall number of SAR data points in pharmaceutical companies was disclosed in the past; some numbers reported in literature are listed in **Table 1**. Although this information is not up-to-date, it can still give a sense of the scale of experimental compound data in pharmaceutical industry.

**Table 1:** Number of SAR data point in large pharmaceutical companies reported in literatures.

Company	# of SAR point	Date	Reference
AstraZeneca	150 million single-shot SAR points, 14 million^a^ CR SAR points	Up to 2008	(Proffitt, 2008; Muresan et al., 2011)
Boehringer Ingelheim	260 million single-shot SAR points, 7 million CR SAR points	Up to 2011	(Beck, 2012)
Pfizer	0.6 million CR SAR points	Up to 2005	(Paolini et al., 2006)
Johnson & Johnson	30 million SAR points	Up to 2006	(Agrafiotis et al., 2007)

a) This number includes external sources, up to 2012.

Comparing with conventional HTS screening with a limited number of data readouts per compound, high-content screening (HCS) (Bickle, 2010) using automated microscopy generates images with multi-parameter readouts that provide an information-rich characterization of cellular phenotypic responses to small molecules. It has become an important tool for compound profiling and has led to a substantial increase in the amount of compound profiling data. For example, 460,800 images were produced through a screen comprising 100 384-well plates imaged with three fluorescent channels at four independent sites per well (Boutros et al., 2015). Hundreds of parameters can be extracted from each cell in the image quantifying information of morphological, geometric, intensity, and texture-based features. Recently Janssen reported (Simm et al., 2018) an image dataset for 524,371 compounds originally used for the detection of glucocorticoid receptor (GCR) nuclear translocation. For each cell in the image, 842 features were extracted, corresponding to roughly 440 million data points. The usage of image-based compound profiling data will be discussed in a subsequent section.

High throughput mRNA expression profiling can be used to characterize the response of cell culture models to perturbations such as small molecules acting as pharmacologic modulators (Lamb et al., 2006; Iorio et al., 2013). These compounds induce transcriptional effects that can be used as gene signatures to discover new connections among compounds, pathways, and diseases. With one of these technologies, known as L1000™ Expression Profiling (profiling for 978 gene expressions) (De Wolf et al., 2016; ), thousands of compounds can be screened per day at lower costs than conventional microarray techniques (Subramanian et al., 2017). Merck reported the screening of a set of 3,699 compounds using the Genometry L1000 platform to unveil a new target for compounds (Filzen et al., 2017). Janssen announced (How library-scale gene-expression profiling is changing drug discovery; Pascale, 2015) that they will use Genometry’s L1000 platform to generate gene-expression profiles for 250,000 compounds from Janssen’s small-molecule screening library. It is expected that more pharmaceutical companies will adopt similar technologies and approaches to generate large-scale transcriptomics data for compound profiling.

With the continuous increase in the amount and heterogeneity of data that are generated and stored in large repositories, the question of how to ensure and sustain data integrity gained more and more attention. The generation and storage of large amounts of data require significant investments in IT infrastructure. These investments are justified not only by efficiency gains for ongoing projects through elimination of manual steps to compile and analyze project-relevant data that ultimately lead to decisions on whether or not to pursue a certain molecule or compound class, but also perhaps even more so by the prospect to discover knowledge across projects as described for example in recent publications by Novartis (Wassermann et al., 2015a) or Boehringer Ingelheim (BI) (Beck, 2012). All this is only possible if the data context is provided alongside the data itself, and when there is a profound understanding of the data quality. One important aspect for consideration is the assay technology that is applied for compound testing. The direct interference of compounds with an assay technology is a source for systematic errors, which should be considered when analyzing the respective data sets. In a recent example at BI (Beck et al., 2015), the screening deck was assayed against an ion channel target for neuroprotection by means of a fluorometric imaging plate reader (FLIPR) assay (Sullivan et al., 1999). The screen yielded a high hit rate, and using a systematic overlap analysis with results from previous FLIPR campaigns, a large number of compounds most likely to be false positives were excluded from labor-intensive follow-up activities. Other important aspects regarding data quality are, for instance, compound purity, autofluorescence, or physicochemical properties such as aggregation propensity (Jadhav et al., 2010), which can have a significant influence on assay results and need therefore to be taken into account as decision-relevant context. This can be accomplished by computational surrogate parameters or auxiliary experiments such as high-throughput solubility determination *via* nephelometry (Fligge and Schuler, 2006).

Typically, data repositories within pharmaceutical companies evolve over years, and the best practices as to which data to store in such systems do so as well. This leads to situations in which legacy data are hardly comparable with present results, thereby limiting the chances to add value from mining data, which were generated at significantly different points in time. Efforts to set up data governance structures and to employ modern technologies around meta data management and central nomenclatures aim to address this issue and are currently underway in many companies (Proffitt, 2008).

## Biological Profiling Descriptors for Hit Expansion

Traditionally, cheminformatic approaches focused on the use of molecular descriptors that are related to structure in order to describe the biological activities of compounds. Among them, structural fingerprints have been intensively used in similarity search, clustering, as well as in building SAR models (Willett, 2011). This is largely based on the hypothesis that structurally similar molecules are likely to bind to the same group of protein and then—as a consequence—share similar biological profiles (Martin et al., 2002; Keiser et al., 2007; Willett, 2011). In the late 1980s, NCI pioneered the implementation of a biological fingerprint to access the similarity of compounds (Paul et al., 1989). In contrast to structural fingerprints, biological activity data are utilized to describe a compound, neglecting structural features. Furthermore, with the recent advent of phenotypic screening, we observe an increasing awareness that the cellular effects of a compound can be described by its interaction with the proteome, without requiring the knowledge of the molecular structure.

**Figure 2:**
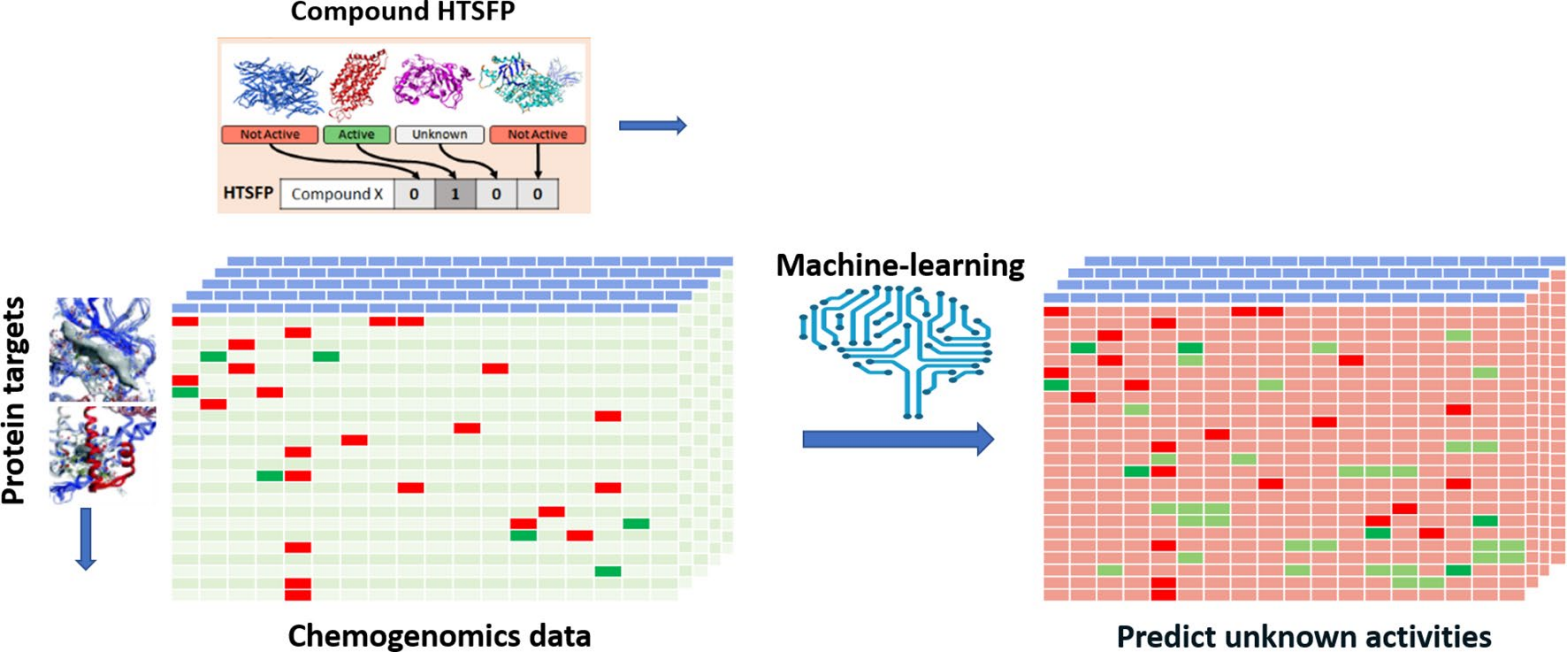
Illustration of applying HTS-FP for building multi-task learning models. A chemogenomic matrix represents the interactions between the compound collection and a panel of biological target. Such a matrix is very often sparsely filled activities and missing cells represent unknown activity for the compound/target pair. Employing machine learning and HTSFP is an example of how unknown activities can be predicted.

Efforts have been devoted to transpose various types of biological responses into fingerprint format that could be used to access biological similarity of ligands (Kauvar et al., 1995; Fliri et al., 2005a; Fliri et al., 2005b; Plouffe et al., 2008; Dixon and Villar, 2010). Recently, researchers of Novartis reported the use of the huge amount of in-house HTS data for this purpose (Petrone et al., 2012). The aggregated data from 195 biochemical and cell-based assays for around 1.5 million of compounds have been employed to generate biological fingerprints, so called *HTS-FP*. They stressed the usefulness in mixing biochemical and cell-based data in detecting molecules that can produce similar phenotype without necessarily presenting the same mode of action (Petrone et al., 2012). They demonstrated the complementarity between the *HTS-FP* and a state-of-the-art molecular fingerprint [e.g., ECFP4 (Rogers and Hahn, 2010)] in similarity searches, especially in relation to the scaffold hopping potential of *HTS-FP* to identify structurally diverse hits. On the other hand, biological fingerprints were found to be more efficient in a study related to screening plate selection and hit expansion (Petrone et al., 2012). Additionally, it was observed that biological fingerprint-based clusters contain compounds that interact with targets that operate jointly in the cell. In further work, the combination of *HTS-FP* with structural fingerprints *via* the use of various machine-learning approaches has showed promising results in HTS hit expansion (Riniker et al., 2014). Other studies showed the usefulness of *HTS-FP* for iterative screening purpose (Paricharak et al., 2016). *HTS-FP* has one major drawback though, which is that predictions cannot be made for compounds that have not been previously tested in any HTS assays. In addition, HTS predominantly produces much more *inactive* than *active*, which consequently leads to quite sparse *HTS-FP*. To tackle these issues, Laufkötter et al. (2019) have developed a method where missing bioactivity data were compensated by considering structural data in a so-called combined fingerprint (CESFP) (**Figure 2**). They reported a significant improvement when using CESFP compared to the use of *HTS-FP* and Extended Circular Fingerprints (ECFP) alone in random-forest based activity prediction models. This indicates a clear synergistic effect between structural and biological fingerprints. *HTS-FP* have also been employed for multitask ML. In a recent study, it was observed that *HTS-FP* and ECFP based activity predictions, while comparable in performance, could return hits containing different chemotypes, suggesting that combining these approaches can be an efficient way to explore the bioactive chemical space (Sturm et al., 2019).

Leveraging the transcriptional data such as gene expression profile (gene signature) in a cell could be another way to construct a biological profile descriptor. The publicly funded CMap database (Connectivity Map; Lamb et al., 2006) initially contained profiles of 164 drugs and later expanded to 1,309 FDA-approved small molecules. These small molecules were tested in five human cell lines, generating over 7,000 gene expression profiles in the database (Lamb et al., 2006). Compound induced gene signature profiles have been used for finding diverse hits (Lamb et al., 2006) and drug repositioning (Ishimatsu-Tsuji et al., 2010; Sirota et al., 2011). Although generating this kind of compound related cell perturbation data is still quite expensive, several pharmaceutical companies, as mentioned earlier, are moving in the direction of generating such data in a large scale. It can be expected that transcriptomics-based biological descriptors will be explored for hit identification in the future. Other biological descriptors derived from multiplexed image data have been reported and successfully used for several tasks, which will be discussed in the subsequent imaging section.

## Analysis of Image-Based Profiling Data With Machine Learning

In the drug discovery process, biological imaging and image analysis are widely used at various stages ranging from preclinical research to clinical trials. Imaging techniques enable the visualization of phenotype and behavior at multiple levels, including full body of humans or animals, organs, tissues, cells, subcellular compartments, and single molecules. A wide range of available imaging techniques can help to reveal the distribution of a drug in the body, organ, and cell as well as its mechanism of action. Such techniques rely on image datasets obtained through automated microscopy. An example of a large-scale image dataset is given by The Cell Image Library (Bray et al., 2017), which contains 919,265 five-channel fields of view related to 30,616 compounds. The most common imaging techniques are automated microscopy using several fluorescent markers as well as label free microscopy such as brightfield and digital phase contrast. These imaging techniques and the downstream data analysis produce a large amount of data and associated extracted features. For several decades, automatic analysis methods (Boutros et al., 2015) have been successfully applied to identify objects such as organs, tissue types, cells, and subcellular compartments. Effects of diseases and drugs could be quantified by applying statistics and ML methods on the features that were extracted from the images in post-processing efforts. However, recent developments in deep NNs and specifically convolutional NNs (CNNs) are revolutionizing the field and setting new gold standards for key tasks such as segmentation and classification (Kraus et al., 2016; Chen et al., 2016; Dürr and Sick, 2016; Kraus et al., 2017). These new methods not only achieve better results but also avoid the time-consuming manual work of designing features and searching analysis methods for specific tasks. To achieve this, relatively large annotated data sets and substantial computational resources as provided in modern GPU clusters are required for training.

**Figure 3:**
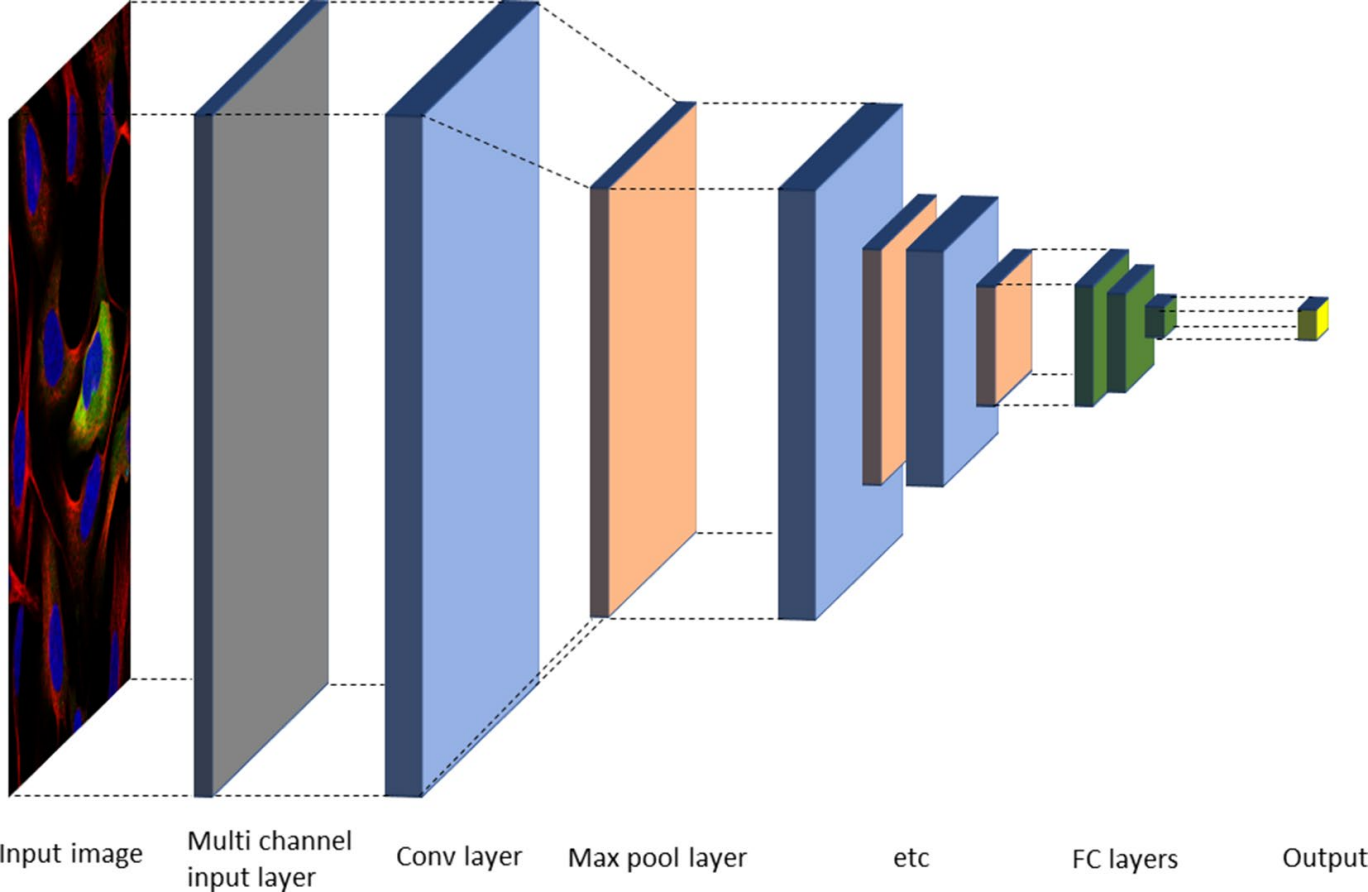
Typical neural network architecture for image classification. Alternating convolutional and max pool layers are followed by a number of fully connected layers, and finally an output layer with either sigmoid or softmax functions, depending on the task (Gawehn et al., 2016).

Deep neural nets (typically CNNs) have now been successfully applied for most tasks occurring in automated cell and tissue microscopy image analysis, including denoising (Su et al., 2015), super resolution (Nehme et al., 2018; Ouyang et al., 2018; Rivenson et al., 2018; Wang et al., 2019), stain normalization (Janowczyk et al., 2017), hit identification (Simm et al., 2018), protein localization (Pärnamaa and Parts, 2017), cell cycle phase classification (Eulenberg et al., 2017), mechanism of action classification (Kensert et al., 2019), focus quality check (Yang et al., 2018), segmentation both in 2D and 3D (often using some version of a U-net architecture (Ronneberger et al., 2015)), and modality estimation (Christiansen et al., 2018). Many tasks fall in the area of classification, including tasks such as quality control (Yang et al., 2018), object detection (Ren et al., 2017; Hung et al., 2018), or outcome classification (Cireşan et al., 2013). Classification can be performed either on the image level or on the object level. In the latter case, it is linked to a localization or detection task to identify objects in a given image. One common two-step approach used is to first select candidate regions and then classify them. Alternatively, the network output consists of a probability map, which is analyzed in a postprocessing step to identify the objects. A typical architecture for classification is shown in **Figure 3**.

Since large amounts of annotated data are often not available for a specific task, strategies such as transfer learning are often applied, e.g., for classification tasks (Kensert et al., 2019; Zhang et al.). This starts with a pretrained neural net from a different task where a large data set is available. The model is then used as an initialization for the new task and fine-tuned for the task at hand. The last output layers of the original network are often not reused but trained for the new task from scratch.

As mentioned above, HCS where cells are exposed to different compounds followed by automated multichannel microscopy and subsequent automatic feature extraction is producing much richer data for screening than traditional HTS. More advanced analysis of cells exposed to chemical perturbations allows to identify related spatial and temporal information. Different biological descriptors derived from multiplexed image data have been reported (Loo et al., 2007; Young et al., 2008; Feng et al., 2009; Caicedo et al., 2017). Reisen et al. (2015) derived a biological fingerprint from HCS. Their HCS fingerprints are based on an automatic analysis of a panel of imaging assays that recorded morphological changes within six different cellular compartments upon testing of 2,725 compounds with well-characterized mode of actions. These fingerprints were then used in classifying the compounds into clusters, which were subsequently annotated with target activities from bioactive molecules from different databases such as ChEMBL, Gostar (), Drug bank (Knox et al., 2011), Integrity (Thomson Reuters), or Metabase (Thomson Reuters). Phenotypic responses were successfully classified for 52% of the tested compounds, and different phenotypes were identified that could be linked to the modulation of individual targets, cellular pathways, or disease genes (Reisen et al., 2015). Later, Simm et al. (2018) built a supervised machine-learning model based on fingerprints obtained from morphological features extracted from high-throughput (cell) imaging (HTI) screening data. Their method enabled the identification of additional hits that were diverse from those obtained in a primary screen. More recently, end-to-end convolutional NNs (Hofmarcher et al., 2019) were used on cell-painting images to predict assay activity as a multitask prediction problem. A number of common architectures were compared to each other as well as to the baseline model constructed with CellProfiler (Carpenter et al., 2006) extracted features. End-to-end models were shown to be able to deliver better results without first extracting features from the images.

## Predicting Compound Activity Using Large Chemogenomics Models

One of the main purposes of chemogenomics (Caron et al., 2001) is to obtain a matrix containing all the possible and impossible interactions between compounds covering the entire chemical space and biological proteins. Despite the advances in HTS (Hertzberg and Pope, 2000) techniques, which made it possible to test hundreds of thousands of compounds against a biological target in very little time, it seems quite unlikely that we will ever obtain a full chemogenomic matrix due to the complexity of the chemical space (Reymond, 2015) and the cost and time such a task would require due to the sheer size of the chemical space. It is, however, possible to computationally predict interactions between chemical compounds and panels of biological targets. The generation of such chemogenomic models is enabled by large databases that contain compounds with annotated biological activities. An applied example of activity predictions relying on chemogenomic models is shown in **Figure 2**. As previously mentioned, a large amount of SAR datapoints from assays with constant conditions and well-characterized quality can be found in private pharmaceutical companies’ databases. In the public domain, the most known databases are ChEMBL (Davies et al., 2015; Gaulton et al., 2016), PubChem (Kim et al., 2019), and BindingDB (Gilson et al., 2015). ChEMBL is a manually curated database of bioactive molecules with drug-like properties. PubChem is a repository for screening data and BindingDB contains affinity measurements data. ChEMBL and BindingDB data were manually extracted from peer-reviewed journal articles. Furthermore, large amounts data from publications and patents are available in commercial databases such as Reaxys (Reaxys Database) and .

A major topic that has been briefly addressed previously is the necessity of data standardization and curation prior to building a predictive model. Chemical structures can be represented by different types of notations (SMILES, InChI, etc.) (InChI and InChIKeys for chemical structures; Weininger, 1988; Weininger et al., 1989; Heller et al., 2015), and bioactivity data typically originate from different assay formats and are reported in a variety of units. One recent example of such a standardization exercise was reported by Sun et al. (2017) and resulted in the creation of a unified dataset, ExCAPE-DB, covering over 70 million SAR data points coming from PubChem and ChEMBL. In another study, Mervin et al. (2015) mined ChEMBL active compounds and PubChem inactive compounds to construct a dataset of 195 million bioactivity data points and investigated the impact of inactive data on the performance of a predictive model.

Several models (Wang et al., 2013; Sushko et al., 2014; Hughes et al., 2016) employing various ML methods or virtual screening are available for target predictions and compound reactivity prediction, but only a few were derived from larger datasets. Studies on small-scale datasets (i.e., on very few assays or targets) can lead to misinterpretation of results or incorrect generalization as their applicability domain is limited. When using small dataset, there is a risk of investigating compounds that do not cover a wide range of the chemical space. In such a scenario, predictive models would show excellent performance when applied on structurally similar compounds but would fail to predict the activity of compounds pertaining to other series. Most compound-target profiles are sparsely filled. One method to compensate missing data is to combine bioactivity data with structural data as we have discussed in the previous section. Applying ML methods on large chemogenomic datasets has been reported in literature. Mervin et al. (2015) constructed a dataset of over 195 million bioactive data points and demonstrated that the inclusion of inactivity data improves the accuracy of predictive models. Another example for modelling large-scale chemogenomic data was reported by Martin et al. (2019) and produced activity predictions as accurate as an experimental 4-concentration IC_50_s. A profile-QSAR (pQSAR) model based on 11,805 Novartis assays was applied on 5.5 million Novartis compounds, leading to a total of 50 billion predictions. This model is updated monthly. Recently, deep learning methods were also applied to build multi-task models. A study by Mayr et al. (2018) applied a variety of ML methods on a dataset of 45,000 compounds contained in more than 1,000 assays extracted from ChEMBL. It was shown that deep-learning outperforms all the other tested methods [i.e., RF (Breiman, 2001), SVM (Cortes and Vapnik, 1995), K-Nearest-Neighbors (Silverman and Jones, 1989), Similarity Ensemble Approach (Keiser et al., 2007), Naïve Bayes (Zhang, 2004) statistics] for target predictions. The strength of this analysis relies on the fact that it was not biased by specific chemical structures or a particular structure representation of the compounds, as the dataset covered a wide range of target families, and various types of fingerprints were employed. This analysis showed that the performance of the predictive model increases with the training set size, confirming that effort should be put into creating large dataset for ML methods. Efforts for estimating prediction uncertainty of ML models have also been reported, for example, conformal prediction framework-based methods (Bosc et al., 2019; Cortés-Ciriano and Bender, 2019) and Bayesian-based approaches (Zhang and Lee, 2019). A study (Tsubaki et al., 2019) employed GNN and CNN to infer protein–compound interaction predictions and determine the importance of each subsequences of the proteins in the interaction. In **Table 2**, we summarized some studies in which DNN has been shown to outperform traditional ML approaches.

**Table 2:** Performances comparison of traditional ML and DL in Drug Discovery.

Ref.	Performance traditional ML	Performance deep-learning
(Koutsoukas et al., 2017) (1)	RF: MCC = 0.89	DNN: MCC = 0.91
(Dahl et al., 2014) (2)	RF: AUC = 0.78	MT NN: AUC = 0.82
(Lenselink et al., 2017)	SVM: MCC = 0.50, BEDROC = 0.88	DNN_MC: MCC = 0.57, BEDROC = 0.92
	RF: MCC = 0.56, BEDROC = 0.82	
(Mayr et al., 2016)	SVM: AUC = 0.71	ST: AUC = 0.72
		MT: AUC = 0.75
(Feinberg et al., 2018)	RF: Pearson = 0.783	GNN: Pearson = 0.822
(Segler and Waller, 2017b)	LR: Acc = 0.86 (reaction prediction)	NN: Acc = 0.92 (reaction prediction)
	LR: Acc = 0.64 (retrosynthesis)	NN: Acc = 0.78 (retrosynthesis)
(Wu et al., 2018) (3)	SVM: AUC = 0.822	GC: AUC = 0.829
(Xiong et al., 2019) (4)	SVM: AUC = 0.792	Attentive FP: AUC = 0.832
(Yang et al., 2019) (5)	RF: AUC = 0.619	FFN: AUC = 0.788
(Ma et al., 2015) (6)	RF: R^2^ = 0.42	DNN: R^2^ = 0.49
(Ramsundar et al., 2017) (7)	RF: R^2^ = 0.428	ST: R^2^ = 0.448
		MT: R^2^ = 0.468

LR, ST, MT, GC, GNN, and FFN refer to Linear Regression, Single- and Multi-Task, Graph Convolution, Graph, and Feedforward Neural Network, respectively. (1) Averaged performance on validation sets over 7 datasets. (2) Averaged performance on test sets over 19 datasets. (3) Performance on a test subset of the Tox21 dataset. (4) Performance on the HIV dataset. (5) Performance on the Tox21 dataset. (6) Averaged performance over 15 datasets. (7) Model performance on a test set.

Although it is crucial to have a sufficient amount of training data to infer target predictions, having high-quality data is also necessary. Indeed, available activity data can be erroneous due to the problematic nature of the compounds (Dahlin et al., 2015) (e.g., reactivity, impurity, aggregation, technology hitters, etc.) or the experimental conditions in which they were tested (concentration, assay technology, plate type, etc.). The integration of such erroneous and heterogenous data can have an impact on predictive models. Various methods have been developed to detect such problematic compound behaviors, the most popular one being the Pan-Assay Interference Substructure (PAINS) filters (Baell and Holloway, 2010). A significant number of compounds that were initially considered as potential leads were found to be false positives. PAINS filters are substructures that were frequently observed among these compounds. It has now become usual to apply these filters when selecting compounds for follow-up studies. However, the PAINS filters were derived from compounds tested in only one specific HTS technology (namely, AlphaScreen) and do not cover the entire chemical space. Thus, these filters should be applied with care (Baell and Nissink, 2018). Stork et al. (2018, 2019) developed the Hit Dexter model to predict frequent-hitter, aggregator, PAINS, dark chemical matter (Wassermann et al., 2015b), and other potential nuisance compounds. The Hit Dexter model is based on a set of extensively tested compounds from PubChem represented by their 2D molecular fingerprints. The Badapple model (Yang et al., 2016) was developed to filter out promiscuous compounds based on a scaffold promiscuity analysis. Such predictive models and substructure filters are crucial for compounds triaging and data accuracy; however, the characteristics of the data under investigation and the aim of the screening project have to be taken into consideration when applying those filters. Promiscuous compounds, while giving rise to possible negative side effects due to their potential interactions with multiple targets, can still be of great interest because of their polypharmacology. In a similar manner, compounds interfering with an assay technology should not be discarded from a drug discovery process but should, however, be tested in a different technology based on dissimilar mechanisms. Sample impurity is another factor to consider regarding promiscuity. If the purity of each sample tested is known, it is easy to filter out everything that did not match the requested quality criterion. If this is not the case, one can use in-house data to detect promiscuous samples in the screening deck (Beck, 2012).

Other criterion to consider in HTS the druglikeness of a compound, which is determined by the compound’s physicochemical (PC) and toxicological properties. Various quality control pipelines created to filter out compounds employ straightforward filtering rules (Hsieh et al., 2015; Zhai et al., 2016), while some other employ ML techniques such as deep-learning (Liu et al., 2019) methods. In pharmaceutical companies and academic institutes, PC filters are tuned depending on the type of compounds found in the chemical libraries (Brenk et al., 2008; Pearce et al., 2006; Cumming et al., 2013). PC properties-based rules ensure that compounds have similar properties to other drugs based on historical data and have a good probability to be synthesizable and non-toxic. Furthermore, structural alerts have been created (Sushko et al., 2012) to flag potential toxic compounds in terms, for example, of mutagenicity (Tennant and Ashby, 1991) or skin sensitization (Barratt et al., 1994).

Very recently, a new consortium of pharmaceutical, technology, and academic partners has launched the “MELLODDY” (Machine Learning Ledger Orchestration for Drug Discovery) project (MELLODDY Consortium| Twitter; Pharma Companies Join Forces to Train AI for Drug Discovery Collectively). The project involves 17 partners from across Europe and receives funding from the EU Innovative Medicines Initiative (IMI) as a public–private partnership. MELLODDY aims to train chemogenomics models across multi-partner (10 pharma companies) datasets while ensuring privacy preservation of both the data and the models by developing a platform using federated learning. It will be interesting to see their efforts regarding data standardization and generation of a large high-quality data set and the results of such an approach.

## Modelling Chemical Reactions From Large-Scale Synthesis Data

**Figure 4:**
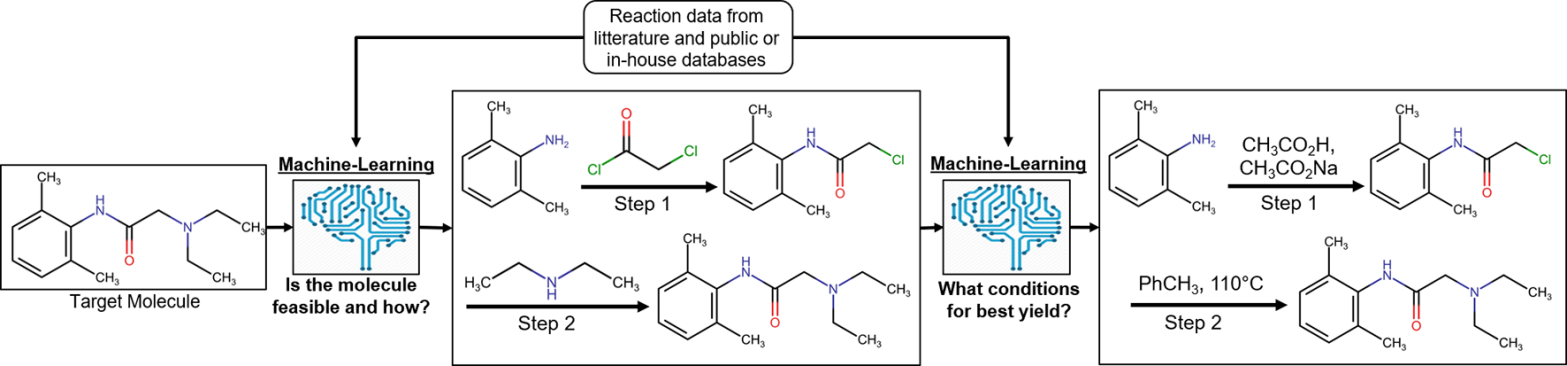
Process of reaction prediction on an exemplary target molecule [lidocaine (Reilly, 2009)]. Machine-learning methods are applied to, first, predict the synthetic feasibility of the molecule and, second, predict the chemical context leading to the best yield possible for the reaction.

It is of crucial importance in drug discovery to be able to predict the feasibility of chemical reactions (Engkvist et al., 2018). It ranges from predicting synthetic feasibility for compounds identified in virtual screening in early drug discovery as well as for hit expansion in the lead generation phase to late stage modifications during lead optimization and to predict possible synthetic routes for upscaling of the synthesis of clinical candidates (**Figure 4**). Synthetic predictions have a long history dating back to rule-based programs in the 1960s (Corey and Todd Wipke, 1969). Several aspects have made reaction informatics a field for active research during recent years. Besides established commercial products with reactions extracted from literature, reaction data have been extracted from electronic laboratory notebooks (ELNs) (Christ et al., 2012) and patents. Schneider et al. (2016) used text-mining to extract 1.15 million unique whole reaction schemes, including reaction roles and yields, from pharmaceutical patents. The reactions were assigned to well-known reaction types such as Wittig olefination or Buchwald–Hartwig amination using an expert system. Also, large-scale reaction data can be generated from high-throughput experimentation. Schematically reaction informatics can be divided into two subfields, retrosynthetic analysis, where a molecule is analyzed and a set of reactions and building blocks are proposed to synthesize the molecule, and forward reaction prediction, where it is predicted if a set of building blocks will react or not and at which conditions a reaction will occur. In recent years, there has been a paradigm shift on how retrosynthesis routes can be predicted. While historically rule-based systems were the most popular method, more recently several studies using ML have shown superior results. One advantage of ML algorithms is that they are generalized methods and not dependent on rigid predefined rules for describing the exact reaction.

In the following, we will focus on recent examples of predicting how to synthesize molecules by mining large corpora of experimental synthesis data. For more general reviews, we refer to recent publications (Warr, 2014; Coley et al., 2018). Segler and Waller (2017b) used reaction fingerprint descriptors to classify reactions. Both hand-coded and automatically extracted reaction rules were used to classify reactions from literature. Three million reactions were classified with the hand-coded rules, while almost 5 million reactions were classified with the automatically extracted reaction rules. Reaction classification models were built with artificial NNs (ANNs). ANNs were found to be superior in predicting reactions than a rule-based system. In another article, they showed that reaction graphs with reactions extracted from literature can be used to predict novel reactions (Segler and Waller, 2017a). A knowledge graph consisting of 14 million molecules was generated, and 8 million reactions and probable novel reactions could be inferenced from. Studies were also published for predicting the reactivity of protecting groups (Lin et al., 2016); 142,000 catalytic hydrogenation reactions were extracted from literature. The reactions were described with condensed graphs of reaction fingerprints. The models showed high accuracy (90%) for predicting optimal conditions for deprotection of protecting groups. The models were also used to identify contradictions in reactivity charts created manually by experts. Coley et al. (2017) developed predictive ML models using 15,000 reactions extracted from US patents. They created a set of candidate reactions based on enumeration of a set of reactants and reaction templates. In a second step, the candidate reactions were described by a set of reaction descriptors, and a NN model was trained to prioritize the candidate reactions. The model predicted the correct reaction in 72% of the cases, the correct reaction was found in 87% of the cases among the top three predicted reactions, and it was found to be among the top five predicted reactions in 91% of the cases. A recent example of predicting reaction conditions with a large data set was published by Gao et al. (2018). They developed a NN model to predict the chemical context [catalyst(s), solvent(s), reagent(s)] and the most suitable temperature for any particular organic reaction. Reactions were extracted from Reaxys and filtered according to various criteria, resulting in ~10 million example reactions. The models were trained on these reactions and were able to propose conditions where a close match to the recorded catalyst, solvent, and reagent was found within the top 10 predictions in 69.6% of the cases. Another noteworthy development in the reaction prediction field is the construction development of a retrosynthesis system using deep learning technologies. Segler et al. (2018b) reported such a system, in which the system reaction DNN models derived from literature reaction data were combined with Monte Carlo Tree Search (MCTS) to identify a set of reactions and building blocks that could be used to synthesize the desired molecule. While most studies have used a reaction template to describe the reaction, it has been shown recently that a template free seq-2-seq approach (i.e., directly translate product SMILES to the predicted reactants in reaction SMILES format) also can give promising results for synthesis prediction (Schwaller et al., 2018a; 2018b). An alternative way of predicting the synthetic pathway exploiting through learned policies has just been published (Schreck et al., 2019).

## Data Driven *De Novo* Molecule Design Through Generative Models and Data Augmentation

Even though industrial compound-bioactivity datasets have millions of data points, many assay results for specific compound series (typical for the lead optimization stage of a drug discovery project) have much less SAR data. However, these datasets can still be augmented and be further exploited with deep learning approaches, such as QSAR and generative modelling. Data augmentation is the process of adding noise or artificial perturbation to the samples in the dataset before training the model in order to make the final models more robust to overfitting (Arús-Pous et al., 2019b). Moreover, in some cases, data augmentation can give additional information to the model. A simple analogy can be found in building image classification models. For instance, a single image with a “dog” will still be recognizable even if it is rotated, cropped slightly, changed in terms of contrast or lightness, etc. Therefore, a single labelled image can be multiplied into multiple training set entries, thus expanding the dataset.

**Figure 5:**
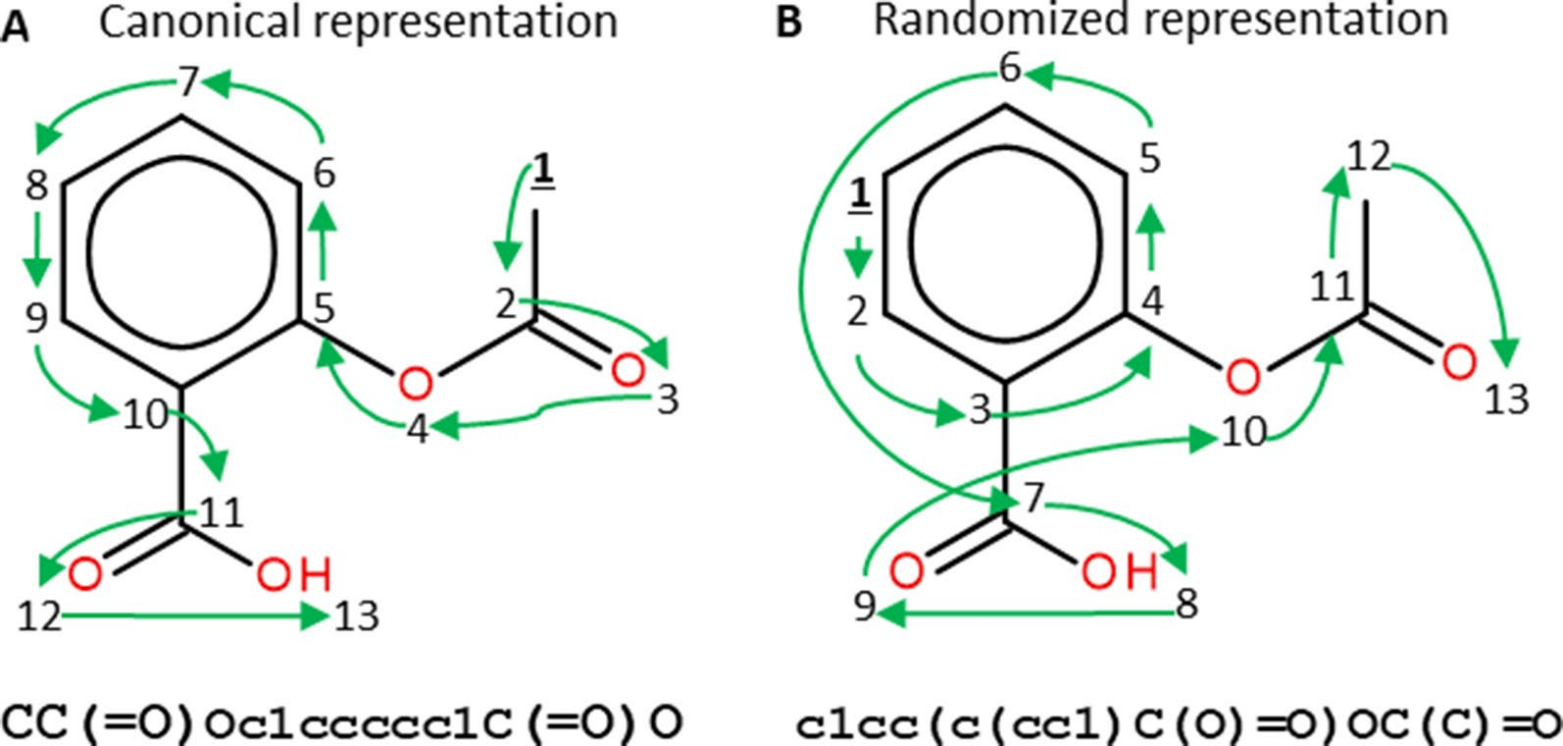
Canonical **(A)** and randomized **(B)** SMILES representations of Aspirin. Numbers represent the atom numberings assigned by the canonicalization algorithm **(A)** or randomized **(B)**. Green arrows indicate how the molecular graph is traversed. Both SMILES strings represent the same molecule but, as the atom numbering changes, the generated SMILES strings do too. Figure extracted with permission from Arús-Pous et al. (2019b).

Similar approaches have also been used in areas relevant to pharmaceutical research such as predicting concentrations of chemical compounds from spectroscopy data (Bjerrum et al., 2017) and building QSAR models from chemical images (Goh et al., 2017). In molecular deep learning models, many architectures use the SMILES as molecular representation (Bjerrum, 2017), which is obtained by assigning a unique number to each atom in the molecule and then traversing the molecular graph using that order. Commonly, a canonical SMILES representation of each molecule is used, which is obtained by calculating a unique numbering for molecules (Weininger et al., 1989). This representation is served as a way of uniquely identifying molecules. Nevertheless, most molecules can have more than one SMILES representation obtained by only changing the numbering of the atoms, meaning that different SMILES start in different atoms of the molecule and traverse it in different ways (**Figure 5**). Randomized SMILES for the same compound can thus be used for data augmentation.

**Figure 6:**
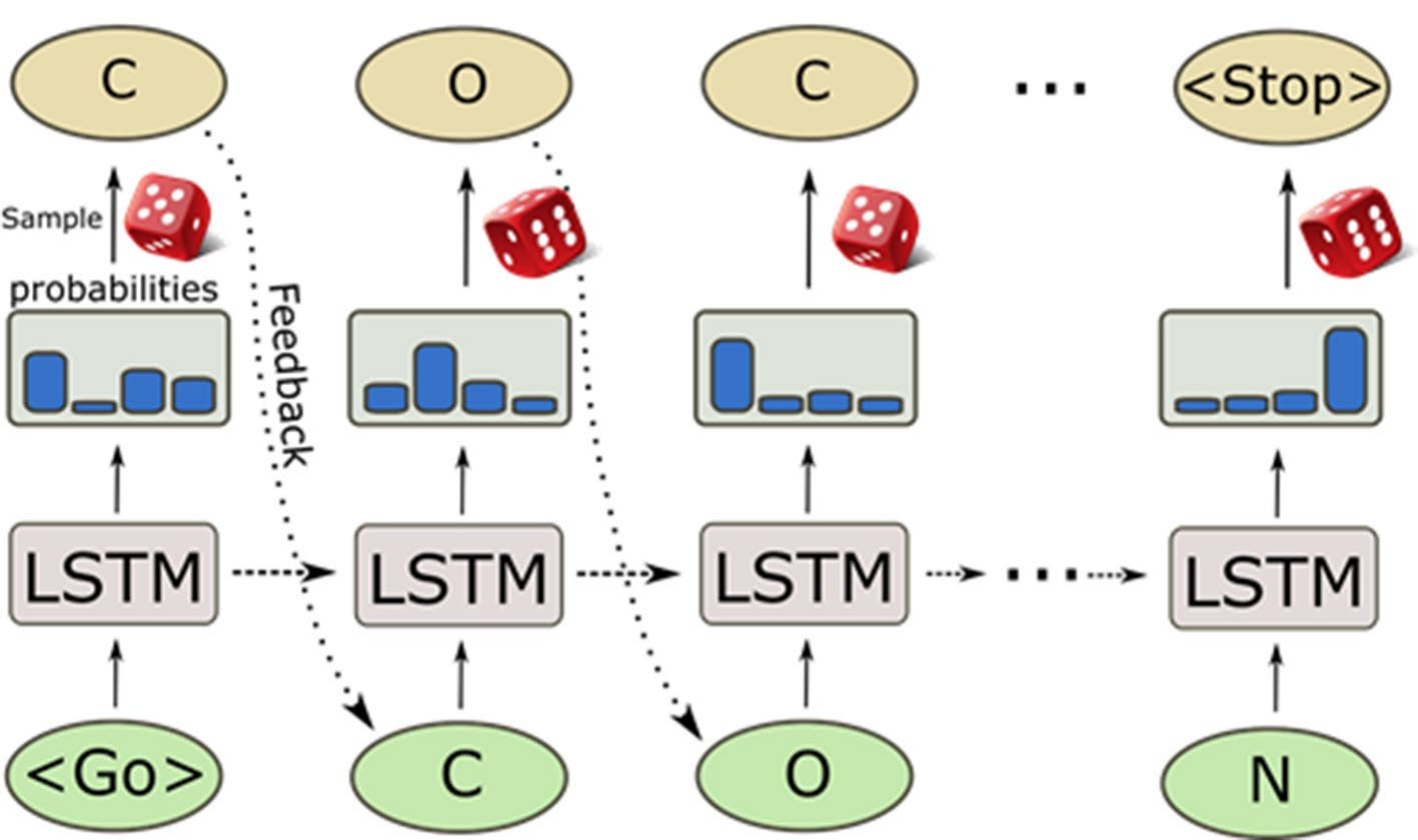
Sampling process of a pre-trained recurrent neural network. The generation process starts with a GO token, and at each step, the model computes a probability distribution of all possible characters. Then, the next character is sampled from it and fed back to predict the next character. The internal memory in the long short-term memory (LSTM) cells enables the predictions to take previous characters into account when generating the next character.

A great surge of interest in cheminformatics applications of deep learning has happened in recent years when NNs were used to generate molecules represented by SMILES strings (Olivecrona et al., 2017; Gómez-Bombarelli et al., 2018; Segler et al., 2018a). Recurrent NN (RNN) trained with a set of SMILES strings can generate molecules that are not present in the training set but that have similar properties as the training samples. These deep learning-based generative models are entirely data driven and do not rely on any predefined reaction/transformation rules, in contrast to the traditional library enumeration methods for generating chemical structures (Schneider and Fechner, 2005). Molecules are generated character by character as SMILES strings by randomly sampling the probability distribution of the next character to sample (**Figure 6**). This process generates a very high ratio of valid SMILES, especially thanks to the use of Long Short-Term Memory (LSTM) (Hochreiter and Schmidhuber, 1997) or Gated Recurrent Unit (GRU) (Cho et al., 2014) cells that capture long-range relationships such as ring closures and branches. Additionally, pre-training on a large set of chemical structures [such as ChEMBL, ZINC (Sterling and Irwin, 2015), etc.] and the subsequent application of transfer learning to smaller datasets can be used to generate focused datasets with an enrichment of active compounds (Segler et al., 2018a). The pre-trained RNNs can also be used to directly optimize toward desirable properties (Olivecrona et al., 2017). This triggered the development of a plethora of novel architectures and techniques in the last years, such as Variational AutoEncoders (VAEs) (Kingma and Welling, 2013; Polykovskiy et al., 2018b; Zhavoronkov et al., 2019), Differentiable Neural Computers (DNCs) (Putin et al., 2018), Generative Adversarial Networks (GANs) (Guimaraes et al., 2017; Prykhodko et al., 2019), and Bayesian optimization method for structure optimization (Pyzer-Knapp, 2018). Besides the SMILES string based *de novo* structure generation methods, algorithms of generating molecules based on molecular graphs have also been proposed and, by using them, methods molecules can be directly generated step-by-step as molecular graphs (Jin et al., 2018; You et al., 2018; Elton et al., 2019; Xu et al., 2019).

Data augmentation techniques have also been applied in molecular generative models. For example, they have shown to improve the quality of the chemical space generated in VAEs (Bjerrum and Sattarov, 2018) and RNNs (Arús-Pous et al., 2019b) in terms of performance of latent vector-based QSAR models (Bjerrum and Sattarov, 2018) and coverage of targeted chemical space (Arús-Pous et al., 2019b). However, there is no consensus on how to measure and compare the performances of generative models. Some approaches have been published, such as MOSES (Polykovskiy et al., 2018a) and Guacamol (Brown et al., 2019), but they are not able to fully characterize the complete chemical space generated. To solve this problem, an approach using the negative log-likelihood (NLL) of generated molecules was recently described (Arús-Pous et al., 2019a). It is able to characterize the models by their completeness, i.e., how many molecules from the target chemical space are sampled, uniformity, i.e., how uniform are those being sampled, and closedness, i.e., how many molecules outside of the target chemical space are being sampled. More specifically, it was found that models trained with 1 million molecules sampled randomly from GDB-13 (Blum and Reymond, 2009), an enumerated database containing 970 million drug-like compounds with up to 13 heavy atoms, are able to generate up to 68% of the entire database when the canonical SMILES representation is used for model training, while the coverage increases to 83%, when non-canonical randomized SMILES are used. It indicates that data augmentation based on randomized SMILES generation has an impact on what models can learn. Moreover, models trained with randomized SMILES generate a much more uniform and closed chemical space than those trained with canonical SMILES.

Deep-learning-based generative model has been applied successfully for prospective design of new druglike molecules with desired activities (Merk et al., 2018). Compounds were generated using a recurrent NN trained on a large set of bioactive compounds. By transfer learning, this general model was fine-tuned on recognizing retinoid X and peroxisome proliferator-activated receptor agonists. The five top-ranking compounds were synthesized and investigated in cell-based assays. Four of these compounds showed a strong affinity toward the targets, with nanomolar to low-micromolar receptor modulatory activity. Generative modelling can also be applied to other chemical entities, such as peptides (Grisoni et al., 2018; Müller et al., 2018), but no method for data augmentation has been described up to now. A potential challenge might be that it is not possible to simply permute the amino acid sequence of peptides as it is done with the arbitrary atom order in SMILES strings, although it may be possible to integrate data from larger unlabelled datasets. PSI-BLAST similarity searching has been used to expand the prior dataset of known active compounds before generation and selection in iterative optimization rounds (Yoshida et al., 2018). This suggests that bioinformatics approaches area a viable way to find the natural variation for the amino acid substitutions and thus enable data set expansion. The drug-like chemical space is estimated to have at least 10^24^ molecules (Bohacek et al., 2010), and it is not feasible to fully enumerate. Nevertheless, deep-learning-based generative models combined with data augmentation techniques have the potential to provide a way to sample large regions of the drug-like chemical space. In combination with synthesis routes prediction, this would deliver a tremendous boost for compound design in pharmaceutical research.

## Conclusion

Over the past years, large amounts of heterogeneous data characterizing the biological action of small molecules have been accumulated in pharmaceutical R&D, stored in both proprietary and publicly available data bases. The origin of these data ranges from biochemical or cellular assays to experiments that investigate the impact of compounds on transcriptomics signatures and assays with imaging readouts. These fast-growing data have fuelled the application of data-savvy ML methods, and in particular deep learning, in order to detect patterns that allow to derive hypotheses for compound-mediated effects on biological (model) systems or to generate predictive models that can be employed at various stages during identification and optimization of new drug candidates. Together with deep-learning-based approaches to sample the drug-like chemical space that—depending on the use case—can be applied with or without predictions of synthetic accessibility, a plethora of potential high-impact applications is emerging. It offers the opportunity to accelerate early drug discovery and to enable a much more comprehensive exploration of the chemical space and the biological effects of its members than traditional wet lab and virtual screening approaches.

## Author Contributions

JMK, BB, and HC wrote the section Large-Scale Compound Data in Pharmaceutical Industry. TK wrote the section Biological Profiling Descriptors for Hit Expansion. JK wrote the section Analysis of Image-Based Profiling Data With Machine Learning. LD wrote the section Predicting Compound Activity Using Large Chemogenomics Models. OE wrote the section Modelling Chemical Reactions From Large-Scale Synthesis Data. JA-P and EB wrote the section Data Driven de Novo Molecule Design Through Generative Models and Data Augmentation. LD and HC co-supervised the manuscript.

## Funding

LD and JA-P have received funding from the European Union’s Horizon 2020 research and innovation program under the Marie Sklodowska Curie grant agreement No 676434, “Big Data in Chemistry” (“BIGCHEM”, http://bigchem.eu). The article reflects only the authors view and neither the European Commission nor the Research Executive Agency (REA) are responsible for any use that may be made of the information it contains.

## Conflict of Interest

Authors LD, JA-P, JK, OE, EB, TK and HC were employed by AstraZeneca. Authors JMK and BB were employed by Boehringer Ingelheim Pharma GmbH & Co. KG.
